# Folic acid prevents cardiac dysfunction and reduces myocardial fibrosis in a mouse model of high-fat diet-induced obesity

**DOI:** 10.1186/s12986-017-0224-0

**Published:** 2017-11-02

**Authors:** Wei Li, Renqiao Tang, Shengrong Ouyang, Feifei Ma, Zhuo Liu, Jianxin Wu

**Affiliations:** 1 Graduate School of Peking Union Medical College, NO. 9, Dongdansantiao, Dongcheng District, Beijing, 100730 China; 2 Department of Biochemistry, Capital Institute of Pediatrics, NO. 2, Yabao Road, Chaoyang District, Beijing, 100020 China

**Keywords:** High-fat diet, Myocardial fibrosis, Oxidative stress, Folic acid, Cardiac function

## Abstract

**Background:**

Folic acid (FA) is an antioxidant that can reduce reactive oxygen species generation and can blunt cardiac dysfunction during ischemia. We hypothesized that FA supplementation prevents cardiac fibrosis and cardiac dysfunction induced by obesity.

**Methods:**

Six-week-old C57BL6/J mice were fed a high-fat diet (HFD), normal diet (ND), or an HFD supplemented with folic acid (FAD) for 14 weeks. Cardiac function was measured using a transthoracic echocardiographic exam. Phenotypic analysis included measurements of body and heart weight, blood glucose and tissue homocysteine (Hcy) content, and heart oxidative stress status.

**Results:**

HFD consumption elevated fasting blood glucose levels and caused obesity and heart enlargement. FA supplementation in HFD-fed mice resulted in reduced fasting blood glucose, heart weight, and heart tissue Hcy content. We also observed a significant cardiac systolic dysfunction when mice were subjected to HFD feeding as indicated by a reduction in the left ventricular ejection fraction and fractional shortening. However, FAD treatment improved cardiac function. FA supplementation protected against cardiac fibrosis induced by HFD. In addition, HFD increased malondialdehyde concentration of the heart tissue and reduced the levels of antioxidant enzyme, glutathione, and catalase. HFD consumption induced myocardial oxidant stress with amelioration by FA treatment.

**Conclusion:**

FA supplementation significantly lowers blood glucose levels and heart tissue Hcy content and reverses cardiac dysfunction induced by HFD in mice. These functional improvements of the heart may be mediated by the alleviation of oxidative stress and myocardial fibrosis.

## Background

The prevalence of obesity is increasing rapidly worldwide [1, 2]. Obesity is regarded as an energy balance disorder with inappropriate expansion and dysfunction of the adipose tissue. Obesity causes a prevalence of type 2 diabetes and cardiovascular diseases that cause left ventricular (LV) hypertrophy, stroke, and heart failure [3, 4]. Moreover, accumulated studies have demonstrated that obesity affects cardiac remodeling with structural and functional abnormalities [5, 6]. However, the underlying mechanisms contributing to these changes remain unclear because of a complex interplay of hemodynamic, neuroendocrine, and metabolic factors causing cardiac hypertrophy, cellular apoptosis, and interstitial fibrosis [7].

Oxidative stress and inflammation have been considered to be involved in the pathogenesis and progression of many forms of cardiovascular diseases [8, 9]. Several studies have indicated that oxidative stress plays a pivotal role in the development of obesity-induced cardiomyopathy [10, 11]. Obesity-related hyperglycemia and metabolic abnormalities cause the production of reactive oxygen species (ROS) in vascular cells and cardiomyocytes [12]. During insulin resistance prior to diabetes onset, ROS accumulation can be found in heart tissue [13], contributing to cardiac dysfunction through lipid peroxidation, extracellular matrix remodeling, mitochondrial damage, and alteration in the coupling proteins [14]. In addition, cardiac fibrosis and inflammation are ameliorated by treatment with antioxidants in the diabetic heart [15, 16]. Therapies aiming to reduce oxidative stress and enhance antioxidant defense have been employed to prevent cardiac dysfunction in cardiovascular diseases [17].

Folic acid (FA) is a B vitamin (vitamin B9) that facilitates the transfer of 1-carbon units in several biosynthetic reactions for two major cellular functions, namely, DNA methylation and the contribution of formyl units to nucleotide synthesis [18]. A meta-analysis of randomized controlled trials has shown that FA supplementation can reduce the risk of cardiovascular diseases [19]. The antioxidant activity is involved in these effects of FA on cardiovascular disorders [20]. Previous studies reported that FA pretreatment blunts myocardial dysfunction during ischemia and ameliorates reperfusion injury and coupled to preservation of high-energy phosphates, reducing ROS generation and ischemia–reperfusion cell death [21].

Hyperhomocysteinemia is associated with a high risk of cardiovascular disease, and dietary folate fortification decreases plasma Hcy levels [22]. The interest in FA for the treatment of cardiovascular disease originates from its critical role in converting Hcy to methionine [23]. FA exhibits an antioxidant effect against ROS and alleviates hyperhomocysteinemia and its associated endothelial dysfunction. Moreover, the anti-inflammatory effect of FA is manifested by a decrease in the levels of some inflammatory mediators in overweight subjects, suggesting a potential therapeutic role of FA in the protection from cardiovascular diseases [24]. Although FA supplementation influences oxidative stress, inflammation, and Hcy metabolism, the direct effect of FA on obesity-related cardiomyopathy has not yet been studied.

In the present study, we hypothesized that FA supplementation in HFD-induced obese mice prevents cardiac dysfunction development by reducing oxidant stress, inflammation, and associated myocardial fibrosis. To test this hypothesis, mice were fed with HFD for 14 weeks in the presence or absence of FA supplementation. Subsequently, mice were measured for weight gain, blood glucose, cardiac function, and heart histology.

## Methods

### Animals and experimental design

Male six-week-old C57BL/6J mice were obtained from Beijing HFK Bioscience Co. Ltd. (Beijing, China) and housed in a temperature-controlled room (23 ± 2 °C) with 12 h light–dark cycle. Mice were provided ad libitum access to food and water. Animals were randomly assigned to a normal diet (ND approximately 10% energy as fat) or a high-fat diet (HFD approximately 60% energy as fat) or an HFD supplemented with folic acid (FAD, 20 μg/mL, Sigma) in drinking water for 14 weeks. The current study used a dose of supplemental FA that is five-times higher than the basal dietary requirements of mice [25]. Body weight was measured weekly. For testing of fasting glucose tolerance, mice were fasted overnight, and then venous blood was drawn by tail clipping. Blood glucose levels were measured with Accu-Chek glucometers (Roche Applied Science, Penzberg, Germany). Cardiac function, morphology, biochemical parameters, myocardial lipid peroxidation, and oxidative stress profiles were assessed when they reached 20 weeks of age. All the procedures performed were in agreement with the Animal Care and Use Committee at the Capital Institute of Pediatrics (CIP 2016032).

### Heart tissue analysis

The heart tissues were homogenized in a 50 mmol/L phosphate buffer (pH 7.2) containing 0.1 mmol/L ethylenediamine tetraacetic acid and centrifuged at 5000×*g* for 10 min for subsequent test. The supernatant of heart tissue homogenates was collected for FA and Hcy analysis. Measurements were performed using a FA and Hcy (Cusabio, Wuhan, China) enzyme-linked immunosorbent assay (ELISA) kit according to the manufacturers’ instructions.

### Echocardiographic examination

After 14 weeks of feeding, all animals were weighed and evaluated by a transthoracic echocardiographic examination. All the measurements were performed by a technician blinded to the experiments. Mice were anesthetized and analyzed using an animal-specific instrument (VisualSonics Vevo770®, Visual Sonics Inc., Toronto, Canada) [26]. LV end diastolic and end-systolic diameters and wall thickness were obtained from M-mode tracings from measurements averaged from six separate cycles. The following structural variables were measured: left ventricular internal diameter at end diastole (LVIDd) and end systole (LVIDs), and LV posterior wall thickness at diastole and systole. Systolic function was assessed based on the LV fractional shortening and LV ejection fraction.

### Histological analysis

After echocardiographic analyses, the mice were euthanized. The heart tissue was also dissected, separated, and weighed. Transverse sections of the LV were fixed in 10% neutral buffered formalin and paraffin embedded. The five-micron-thick paraffin sections were then stained with hematoxylin and eosin (HE). Masson’s trichrome was used for detecting the collagen deposition in the heart tissue. The degree of heart damage was examined blindly using a Leica DMRB/E light microscope (Leica Microsystems, Heerbrugg, Switzerland). The heart muscle and vascular smooth muscle were stained pink, whereas the collagen was stained blue.

### Oxidative stress analysis

The heart tissue was used to assess the activity of antioxidant enzymes, superoxide dismutase (SOD) and catalase (CAT), cellular content of antioxidant glutathione (GSH), and lipid peroxidation [malondialdehyde (MDA)]. The levels of SOD, CAT, and GSH enzyme content were measured using a commercialized assay kit (Beyotime Biotechnology, Beijing, China) according to the manufacturer’s instructions. Protein contents were determined by bicinchoninic acid methods using a commercial kit (Solarbio, Beijing, China). Enzyme activities were normalized to protein concentration in the samples.

### Statistical analysis

All data sets are presented as the mean ± standard error of the mean (SEM). Comparison between the two groups was performed using unpaired two-tailed Student *t*-test using Prism 6.0 GraphPad software (GraphPad, San Diego, CA, USA). A value of *p* ˂ 0.05 is considered statistically significant.

## Results

### FA decreased blood glucose and prevented heart enlargement in HFD mice

**Fig. 1:**
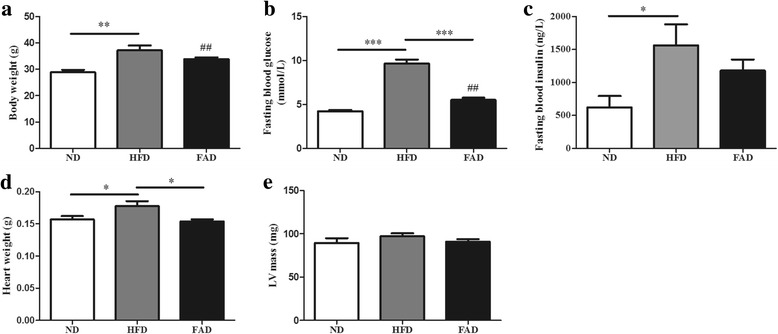
Effects of folic acid (FA) supplementation on body weight, fasting blood glucose, and organ weight of mice fed with a HFD. **a** Body weight; (**b**) fasting blood glucose; (**c**) fasting blood insulin; (**d**) heart weight; and (**e**) LV mass. Values are presented as mean ± SEM (*n* = 6). ND, normal diet; HFD, high-fat diet; FAD, HFD supplemented with FA; LV, left ventricle. *p < 00.05; **p < 0.01; ***p < 0.001. ##*p* < 0.01 versus ND

To characterize the effects of HFD feeding on phenotype, we examined bodyweight, heart weight, and fasting blood glucose levels. No difference was observed in the mean daily dietary intake among the three groups. Body weight significantly increased after 14 weeks of HFD feeding (Fig. 1a, *p* ˂ 0.01). FA supplementation decreased HFD-induced weight gain. However, this difference did not achieve statistical significance (Fig. 1a). Furthermore, we found a significant increase in fasting blood glucose levels in HFD compared with ND, and treatment with the FA markedly decreased blood glucose levels in HFD fed mice (Fig. 1b, *p* ˂ 0.001), but not back to normal level (Fig. 1b, *p* ˂ 0.01). In addition, the serum insulin levels were 2.5-fold higher in HFD compared with ND mice, however, there were no significant differences in serum insulin levels between FAD and HFD mice (Fig. 1c, *p* ˂ 0.05). Homeostatic model assessment of insulin resistance revealed significant insulin resistance in HFD fed mice when compared with the ND groups, and FAD treatment improved insulin sensitivity (data not shown). HFD caused a significant increase in heart weight, and FA supplementation reduced heart weight gain in HFD mice (Fig. 1d, *p* ˂ 0.05). However, LV mass assessment using ultrasonography showed no difference in the LV weight among the three groups (Fig. 1e).

### FA supplementation reversed Hcy elevation induced by HFD

**Fig. 2:**
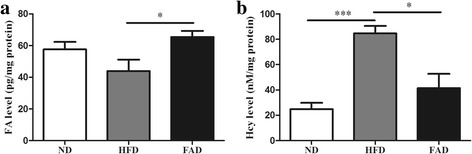
Levels of FA (**a**) and Hcy (**b**) detected in heart tissue by ELISA. Values were presented as mean ± SEM (*n* = 6). ND, normal diet; HFD, high-fat diet; FAD, HFD supplemented with FA; FA, folic acid; Hcy, homocysteine. *p < 0.05; ***p < 0.001

FA deficiency in serum was associated with the risk of cardiovascular diseases [27]. Therefore, we used tissue FA concentrations as an index of methyl donors in heart tissue extracts. No difference was observed in the FA levels between ND and HFD mice. However, the levels of tissue FA were significantly higher in the FAD group compared with the HFD groups (Fig. 2a, *p* ˂ 0.05). We measured heart tissue Hcy levels because FA increases the level of 5-methylene tetrahydrofolate reductas, causing an increase in Hcy metabolism [23]. HFD feeding significantly increased Hcy concentrations in heart tissue (Fig. 2b, *p* ˂ 0.001). Interestingly, we found that FA supplementation reduced Hcy levels in the heart tissue of HFD mice, but there were no significant differences in Hcy levels between FAD and ND mice (Fig. 2b, *p* ˂ 0.05).

### HFD-induced cardiac dysfunction was improved by FA treatment

**Fig. 3:**
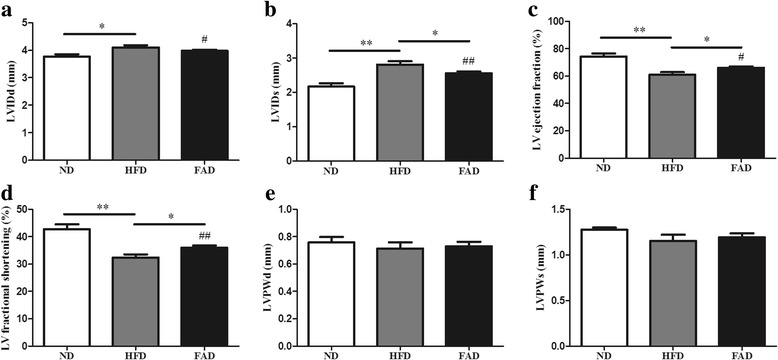
Morphological and functional data evaluated by echocardiography. **a** LVIDd; **b** LVIDs; **c** LV ejection fraction; **d** LV fractional shortening; **e** LVPWd; **f** LVPWs. Values were presented as mean ± SEM (*n* = 5). ND, normal diet; HFD, high-fat diet; FAD, HFD supplemented with FA; LV, left ventricle; LVIDd, LV internal diameter at end diastole, and end systole (LVIDs); LVPWd, LV posterior wall thickness at diastole and systole (LVPWs). *p < 0.05; **p < 0.01. #*p* < 0.05; ## *p* < 0.01 versus ND

Using 2-D M-mode ultrasonography/echocardiography, we observed the development of cardiac dilatation in mice fed HFD for 14 weeks as determined by LVIDd. LVIDs was significantly high in the HFD groups. LVIDs was significantly decreased in FA treatment mice, but not back to ND mice (Fig. 3a and b, *p* ˂ 0.05). We also observed a significant cardiac systolic dysfunction when mice were subjected to HFD feeding as indicated by a reduction in LV ejection fraction and fractional shortening (Fig. 3c and d, *p* ˂ 0.01). HFD mice demonstrated that prominent and significant cardiac dilatation and systolic dysfunction compared with ND mice. Although these alterations were relieved by FA treatment, the FAD mice were unable to return to normal level (Fig. 3c and d). The obesity-induced cardiac dilatation and cardiac dysfunction were improved in mice treated with FA (Fig. 3c and d, *p* < 0.05). However, no difference was found in the LV posterior wall thickness during diastole and systole (Fig. 3e and f).

### FA supplementation ameliorated myocardial fibrosis induced by HFD

**Fig. 4:**
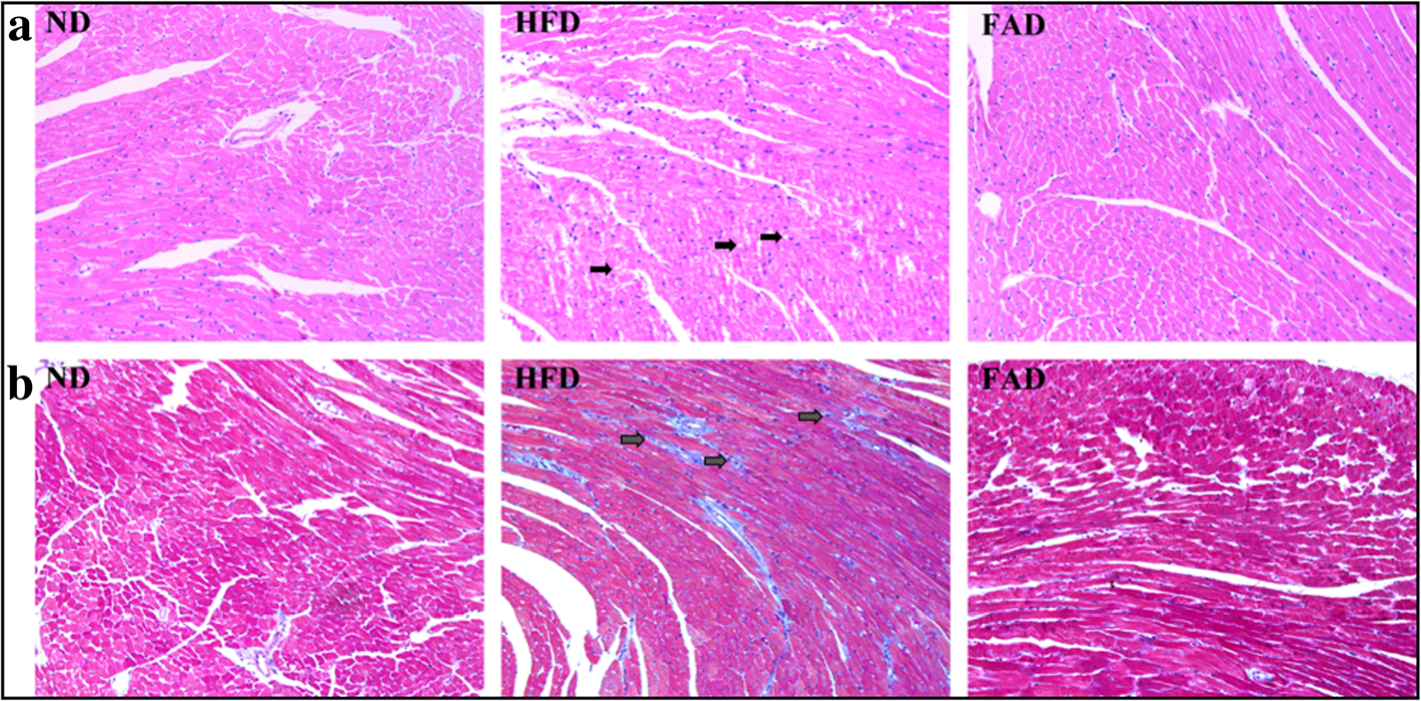
Heart histology after HE staining (**a**) and Masson’s trichrome staining (**b**) of sections from each group (original magnification 200×, *n* = 5). ND, normal diet; HFD, high-fat diet; FAD, HFD supplemented with FA; black arrow, fatty degeneration; gray arrow, collagen accumulation

To investigate further the cardioprotective effects of FA supplementation, we examined the FA effects on the morphology of the heart. HE staining showed that the hearts of HFD-fed mice displayed structural abnormalities and significantly increased fatty degeneration of cardiomyocyte, whereas those alternations of HFD-fed mice were relieved by FA treatment (Fig. 4a). However, no obvious inflammation changes were observed among the three groups. Further staining with Masson’s trichrome revealed a significant increase in collagen accumulation and fibrosis in the hearts of HFD-fed mice. Treatment with FA markedly reduced the degree of collagen deposition and fibrosis (Fig. 4b).

### FA treatment attenuated myocardial oxidative stress in HFD-fed mice

**Fig. 5:**
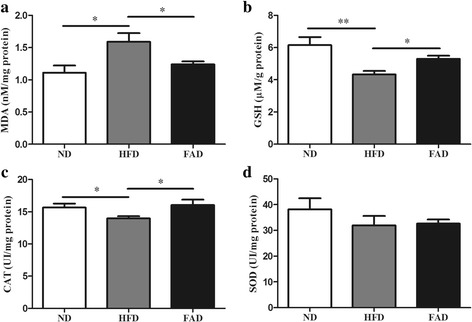
Effects of FA supplementation on oxidative stress in the heart of mice fed with HFD. **a** MDA; (**b**) GSH; (**c**) CAT; and (**d**) SOD levels. Values are presented as mean ± SEM (*n* = 5–6). ND, normal diet; HFD, high-fat diet; FAD, HFD supplemented with FA. *p < 0.05; **p < 00.01

Oxidative stress is associated with cardiac dysfunction in models of obesity-related cardiomyopathy [10]. To evaluate the role of FA in oxidative stress, heart redox status of related biomarkers was determined. Analysis of lipid peroxidation by MDA in the myocardium revealed a significant increase in the HFD group. FA supplementation significantly reduced the levels of MDA compared with the HFD group (Fig. 5a, *p* ˂ 0.05). In addition, HFD consumption obviously caused oxidative stress in the heart tissue as evidenced by significantly reduced the most important antioxidant enzymes GSH and CAT (Fig. 5b and c, *p* < 0.05). However, GSH concentration was increased in mice treated with FA compared with the HFD-fed group but not with the SOD (Fig. 5d). Furthermore, FA treatments restored the concentration of CAT activity to values close to that observed in the ND group (Fig. 5c, *p* < 0.05).

## Discussion

The objective of this study was to evaluate the effects of FA supplementation on the HFD-induced obese mice heart and the role of oxidative stress in this scenario. In the current study, mice fed with HFD exhibited greater weight gain compared with ND mice. Glucose and insulin levels were significantly higher in the HFD group, which is indicative of insulin resistance and consistent with the development of type 2 diabetes. FA supplementation reduced fasting blood glucose levels and improved insulin resistance. Furthermore, we demonstrated that FA supplement ameliorated cardiac dysfunction in HFD-induced obese mice. This result was accompanied with decreased cardiac oxidative stress, fibrosis, and tissue Hcy content.

HFD is related to increasing obesity and heart disease, particularly systolic dysfunction, which characterizes early obesity/metabolic cardiomyopathy [28]. Previous experimental evidence have reported the association between obesity and morphological and functional changes in the hearts of both humans and animals [29–31]. Cardiovascular complications in obesity and insulin resistance often manifest as decreased fractional shortening and impaired cardiomyocyte function [32]. In the present study, HFD feeding induced significant cardiac systolic dysfunction, accompanied with significantly increased LVIDd and LVIDs. However, FA supplementation improved LV fractional shortening and LV ejection fraction in HFD mice. This finding indicates that FAD prevents obesity-related cardiac dysfunction in HFD mice.

Multiple pathways have been implicated in the pathology of obesity-related cardiac dysfunction including, LV fibrosis, increased oxidant stress, inflammation, and alterations in collagen content [33]. Studies have repeatedly shown that oxidative stress and myocardial fibrosis are linked to impaired cardiac systolic function in mouse models of obesity and insulin resistance [34, 35]. In addition, enhanced fatty acid oxidation leads to an overproduction of ROS [36], a byproduct of lipid peroxidation, which plays an important role in increased oxidative stress, as well as diminished energy for myocardial function [37]. Oxidative stress induces several deleterious cardiac alterations including mitochondrial dysfunction, DNA damage, metalloproteinase activation, cellular dysfunction, myocardial fibrosis, and cardiac hypertrophy [38].

Oxidative stress occurs when an imbalance occurs between ROS production and antioxidant systems. Antioxidant enzymes, such as CAT, SOD, and GSH, form the first line of defense against ROS, and the decrease in their activities contribute to the oxidative damage on the tissue [39]. Activities of antioxidant enzymes GSH and CAT decreased in HFD-induced obese mice compared with ND groups and increased in the lipid peroxidation (MDA) in the present study corroborating well with previous studies [40, 41]. FA as a free radical scavenger, if present in physiological concentration, displays potent antioxidant effect, which can protect from free radical damage both directly and via competitive inhibition of xanthine oxidase [42]. The antioxidant activity is thought to be involved in the effects of FA on cardiovascular disorders [20]. Furthermore, the salutary effects of oral FA treatment on endothelial function are partly mediated by an improvement in vascular BH4 bioavailability, which leads to restoration of eNOS coupling and a reduction of eNOS-derived superoxide production [43]. In the present study, we used a dose of supplemental FA that is five times higher than the basal dietary requirements of mice. We found that the FAD group showed higher CAT and GSH content compared with HFD mice, suggesting an improvement in antioxidant defenses.

Hyperhomocysteinemia is recently recognized as an important independent risk factor for cardiovascular diseases [44]. Previously study suggested that hyperhomocysteinemia caused coronary arteriolar remodeling, myocardial collagen deposition, and diastolic dysfunction in hypertensive rats after 10 weeks of dietary intervention [45]. The detrimental effect of Hcy can be alleviated by FA supplementation with a concurrent reduction of superoxide generation [22]. The metabolism of FA and Hcy is interrelated, and increasing FA intake augments Hcy remethylation, leading to a reduction in its plasma concentration, suggesting that treatment with FA may prevent cardiovascular risk by reducing Hcy [46]. Therefore, the beneficial effect of FA on cardiac function may be confer more credit this interrelation. Folate provides the single-carbon moiety in the synthesis pathway for S-adenosylmethionine, which is the main cellular methyl donor that affects methylation reactions. Supplementation with FA alters global DNA methylation profiles [47]. Thus, FA supplementation may allow post-translational modifications of proteins, which could protect contractile function of the heart. Furthermore, FA has been studied in clinical trials, particularly to test its potential to decrease cardiac dysfunction risk in patients with cardiovascular diseases [48]. Recent study shows that FA mitigated cardiac dysfunction by normalizing the levels of tissue Hcy-metabolizing enzymes in myocardial infarction mice [49]. This study emphasizes the importance of Hcy metabolism and FA supplementation in cardiovascular diseases. Previous studies demonstrated that FA ameliorates Hcy-induced oxidative stress [50]. We showed that FA mitigates the cardiac dysfunction in HFD feeding mice. Improvements in oxidant stress and myocardial fibrosis in the heart play a role in this protection. However, further studies are necessary to elucidate the underlying mechanisms.

## Conclusions

The present study demonstrates that the supplement with FA significantly decreases blood glucose levels, reduces heart tissue Hcy content, and protects against obesity-related cardiac dysfunction induced by HFD in mice. The morphological and functional improvements of the heart may be mediated by the alleviation of lipid peroxidation and myocardial fibrosis. These observations imply that FA supplementation inhibits oxidative stress, and thus, may serve as a potential approach to prevent obesity-related cardiomyopathy.

## Abbreviations

CAT: Catalase
FA: Folic acid
FAD: HFD supplemented with folic acid
GSH: Glutathione
Hcy: Homocysteine
HE: Hematoxylin and eosin
HFD: High fat diet
LV: Left ventricular
MDA: Malondialdehyde
ND: Normal diet
ROS: Reactive oxygen species
SOD: Superoxide dismutase
